# The regulation of thermal stress induced apoptosis in corals reveals high similarities in gene expression and function to higher animals

**DOI:** 10.1038/srep30359

**Published:** 2016-07-27

**Authors:** Hagit Kvitt, Hanna Rosenfeld, Dan Tchernov

**Affiliations:** 1 Marine Biology Department, The Leon H. Charney School of Marine Sciences, University of Haifa, Mount Carmel, Haifa 31905, Israel; 2 The Interuniversity Institute for Marine Science P.O.B 469, Eilat 88103, Israel; 3 Israel Oceanographic and Limnological Research, National Center for Mariculture, P.O.B. 1212, Eilat 88112, Israel

## Abstract

Recent studies suggest that controlled apoptotic response provides an essential mechanism, enabling corals to respond to global warming and ocean acidification. However, the molecules involved and their functions are still unclear. To better characterize the apoptotic response in basal metazoans, we studied the expression profiles of selected genes that encode for putative pro- and anti-apoptotic mediators in the coral *Stylophora pistillata* under thermal stress and bleaching conditions. Upon thermal stress, as attested by the elevation of the heat-shock protein gene HSP70’s mRNA levels, the expression of all studied genes, including caspase, Bcl-2, Bax, APAF-1 and BI-1, peaked at 6–24 h of thermal stress (hts) and declined at 72 hts. Adversely, the expression levels of the survivin gene showed a shifted pattern, with elevation at 48–72 hts and a return to basal levels at 168 hts. Overall, we show the quantitative anti-apoptotic traits of the coral Bcl-2 protein, which resemble those of its mammalian counterpart. Altogether, our results highlight the similarities between apoptotic networks operating in simple metazoans and in higher animals and clearly demonstrate the activation of pro-cell survival regulators at early stages of the apoptotic response, contributing to the decline of apoptosis and the acclimation to chronic stress.

Global warming and ocean acidification have recently emerged as key threats to the long-term survival of coral reefs^1^. The oceans’ rapid warming has been held responsible for an increase in the frequency and intensity of mass bleaching events (i.e., the disconnection of the symbiotic zooxanthellae- coral association)^1^, contributing to the high rate (33%) of coral mortality worldwide^2^. Likewise, the continuous acidification of the oceans has caused significant decreases in the concentration of carbonate ions and compromised the ability of marine calcifiers to precipitate calcium carbonate^3^. However, some coral species are known to survive and recover from bleaching^4^,^5^ and reduced pH conditions^6^,^7^. It was postulated that throughout their evolution, corals have survived bleaching and mass extinction events, at least in part due to their ability to switch between soft bodies and calcified fossilizing forms^6^,^7^,^8^,^9^,^10^,^11^. Furthermore, it was suggested that controlled apoptotic response to both thermal stress and reduced pH provides an essential mechanism that enables the coral to counter and survive changing environmental conditions^4^,^7^,^12^,^13^,^14^,^15^.

Apoptosis is a specific form of a highly conserved programmed cell death mechanism that is essential for normal development and tissue homeostasis. The well-studied vertebrate apoptotic machinery displays an intricate network of interactions between the pro- apoptotic and anti-apoptotic mediators, which give rise to death and survival signals, respectively^16^,^17^,^18^,^19^. Apoptosis can be triggered by different stimuli, enabling the activation of caspases (cysteine aspartate-specific proteases) that bring about the orderly destruction of the cell. Members of the Bcl-2 protein family are key regulators of apoptotic cell death in mammals; some prevent apoptosis (e.g., Bcl-2), whereas others are pro-apoptotic (e.g., Bax). The proteins within this family interact in order to govern the permeabilization of the outer mitochondrial membrane, resulting in a release of cytochrome C (Cyt-c), which binds to the protein APAF-1 (Apoptotic Protease-Activating Factor 1) and ultimately activates the caspases (e.g., caspase-3,-9). The fine-tuned control of cell death is further regulated through the inhibitor of apoptosis protein (IAP) family (e.g., survivin gene), as well as through the anti-apoptotic Bax inhibitor-1 (BI-1) (Fig. 1A).

Recent studies indicate the presence of complex repertoires of apoptotic proteins in simple metazoans, with many mammalian counter parts^13^,^20^,^21^. In corals, the expression profiles of apoptotic mediators have been studied under both thermal stress and reduced pH conditions. These studies indicate elevation in caspase activity^4^,^7^,^12^,^14^ and gene expression^4^,^15^, as well as the up-regulation of both putative pro-apoptotic (Bax, Bad^13^) and anti-apoptotic (Bcl-2^4^,^13^,^14^,^15^, BI-1 and BIR^13^) mediators during the first hours/days of stress, followed by a decrease in apoptotic activity and gene expression. Based on these results, it is assumed that the corals’ apoptotic response is divided into two phases: (i) an acute pro-apoptotic response to environmental stress, alongside a rapid activation of antioxidant/anti-apoptotic mediators that block the progression of apoptosis to other cells, and (ii) the termination of apoptosis and the acclimation of the coral to the chronic stress^4^,^7^,^12^,^14^,^15^. Indeed, the experimental data imply that under both thermal stress and reduced pH conditions the allegedly anti-apoptotic coral Bcl-2 members play an important role in regulating apoptosis^4^,^13^,^14^,^15^. However, the functional characterization of these molecules, identified from basal metazoans, is still limited^20^,^21^. To better understand how apoptosis is regulated in corals under stress, we first studied the expression profiles of selected genes that encode for putative pro- and anti- apoptotic mediators in the stony coral *Stylophora pistillata* under thermal stress and bleaching conditions. These genes included HSP70, caspase, Bcl-2, Bax, APAF-1, BI-1 and survivin, reported as key regulators of apoptosis^4^,^13^, and exhibiting significant changes in expression at temperatures below the bleaching threshold^13^. Then, to facilitate support of the hypothesized anti-apoptotic involvement of coral Bcl-2 proteins in down-regulating the apoptotic response, we functionally characterized (both qualitatively and quantitatively) the *S. pistillata* Bcl-2 protein member.

## Results and Discussion

### The identification and expression of target genes that encode for apoptotic mediators in *S*. *pistillata*

**Figure 1:**
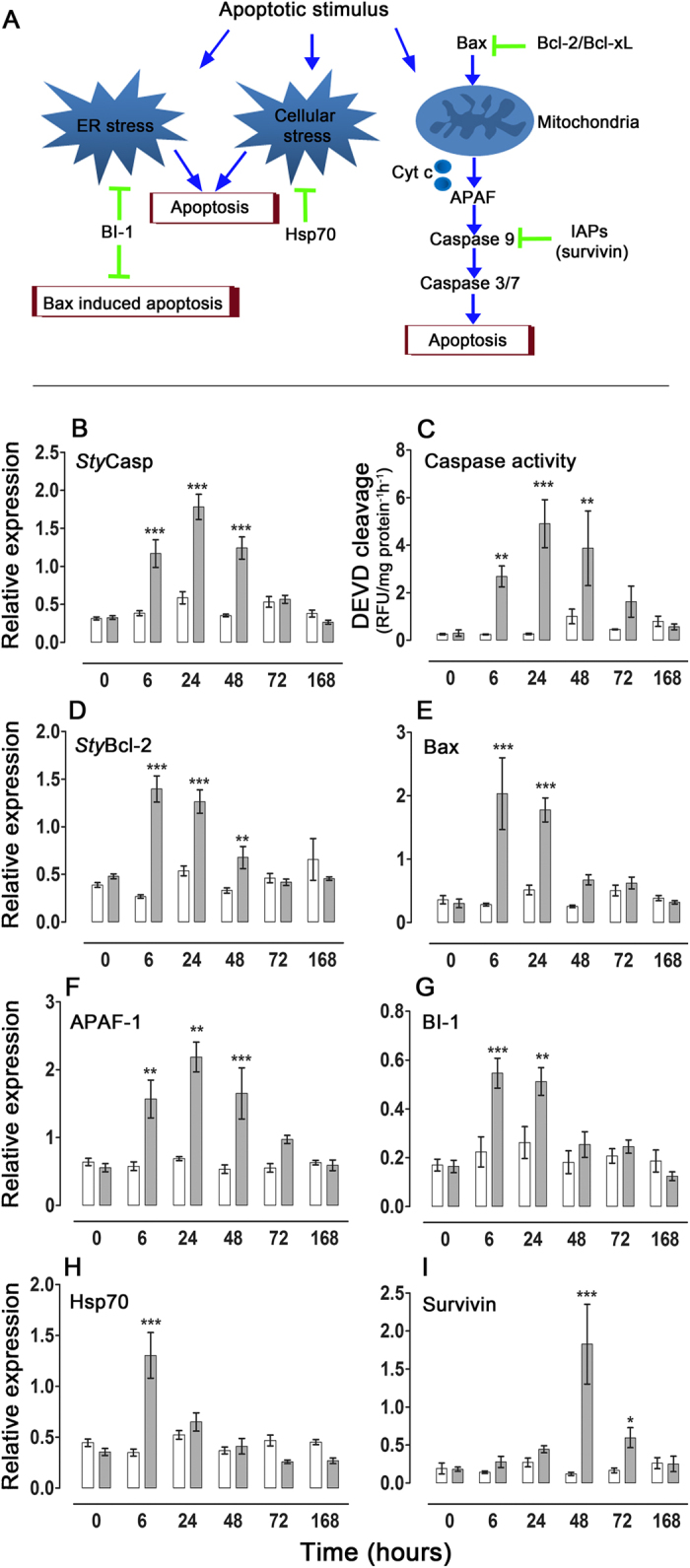
Caspase activity and the quantitative analysis of key regulators of apoptosis. (**A**) A schematic representation of apoptotic networks in mammalians that shows only key regulators relevant for this study. (**B**–**I**) Fragments of *S*. *pistillata* were placed in two aquaria (control at 24 °C and thermal stress at 34 °C). Six fragments from each aquarium were sampled throughout 168 h (n = 6). Results are expressed as the means ± SE of independent extractions from distinct fragments incubated under control conditions (white) or subjected to thermal stress (gray). (**B**,**D**–**I**) A quantitative analysis of key regulators of apoptosis expression normalized to adenosyl-homocysteinase (AdoHcyase). (**C**) Caspase 3-like activity assayed by the fluorometric method using Ac-DEVD-AFC, measured in relative units of fluorescence (RFU’s) and expressed as RFU/μg protein^−1^ h^−1^. Values were tested using a Two-Way Anova with Tukey post-hoc testing. Asterisks indicate significant differences between the control and the thermal stress of the same time point (*represents P < 0.05, **represents P < 0.01, and ***represents P < 0.001). ER, endoplasmic reticulum; Cyt c, cytochrome C.

To better understand the mechanism that underlies apoptotic response in corals and enables them to withstand thermal stress and bleaching^4^, the expression profiles of selected genes that encode for putative pro- and anti- apoptotic mediators were studied in the stony coral *S*. *pistillata* after it was subjected to extended thermal stress for a duration of 168 h (Fig. 1). The expression profiles of the seven studied genes varied in the thermal-stressed *S*. *pistillata* significantly compared to the controls, indicating the possible role of these genes as mediators of apoptosis in corals (Fig.1B, D–I and Table 1). Six of the genes, namely *Sty*Casp, *Sty*Bcl-2, Bax, APAF-1, BI-1 and HSP70, attained maximal levels within 6–24 h of thermal stress (hts), followed by a decline to basal levels within 72 hts (Fig. 1B, D–H). Distinctly, the expression levels of the survivin gene attained maximal levels at 48 hts and were still significantly (P < 0.05) over-expressed at 72 hts (Fig. 1I).

In analogy with the well-studied mammalian apoptotic networks^16^,^17^,^18^ (Fig.1A), the largest gene expression elevation was observed during the first hours of stress for both Bax (Fig.1E) and *Sty*Bcl-2 (Fig.1D) which encode for putative pro- and anti-apoptotic mediators, respectively. Yet, the stimulated expression profiles of Bax and *Sty*Bcl-2 differed in their amplitude, showing a 7- fold and 5- fold increase, respectively, at 6 hts. They also differed in their duration (24 hts for Bax and 48 hts for *Sty*Bcl-2), supporting a “two-stage” response model that could also involve a short-term acute (6–24 h) vs. prolonged moderate-intensity (6–48 h) response.

In metazoans, the Bcl-2 protein family acts as a critical checkpoint for apoptotic cell death, regulating the permeability of the outer mitochondrial membrane^16^. Bcl-2 is also an important regulator of oxidative stress^22^. The balance between anti-apoptotic and pro-apoptotic genes within a cell is critical in determining the ultimate apoptotic rate; the greater the expression level of the pro-apoptotic genes, the more likely it is that the cell will die^17^. In fact, anti-apoptotic and pro-apoptotic gene modulation in the same tissue was reported in clinical conditions and could be indicative of tissue integrity (e.g., over-expression of anti-apoptotic Bcl-2 and Bcl-xL vs pro-apoptotic Bax and Bad)^23^.

Therefore, the over-expression of putative anti-apoptotic/anti-oxidant *Sty*Bcl-2 in the thermal-stressed corals at 48 h could tip the life/death balance towards cell/tissue survival, as suggested in previous studies^4^,^14^. These lines of evidence further support the analogy between coral and human apoptotic networks.

Additionally, the stimulated expression (3.2 fold increase) of the HSP70 gene at 6 hts (Fig. 1H) may also be part of the coral’s anti-apoptotic/anti-stress response. This protein is known to perform its anti-apoptotic action by interacting with endoplasmic reticulum stress sensor proteins and by blocking the recruitment of caspase 9, and also by interacting with protein folding and preventing protein aggregation^24^. Indeed, an up-regulation of HSP70 is documented during the acclimation process of corals to thermal stress^25^ and reduced pH conditions^15^.

The fine-tuned control of cell death in multi-cellular organisms is further regulated through downstream inhibitors (Fig.1A). Our results indicated coordinate expression profiles for both the anti-apoptotic Bax inhibitor-1 (BI-1) and the Bax genes (Fig. 1G,E), with a significant elevation at 6–24 hts (a 2.4 increase for BI-1 and a 1.9 fold increase for Bax). BI-1 is a downstream inhibitor of Bax (Fig.1A) and its anti-apoptotic role is highly conserved throughout both the plant and the animal kingdoms. In addition to its fundamental importance for tissue homeostasis and the regulation of cellular stress, BI-1 is capable of interacting with a broad range of factors in order to inhibit many facets of apoptosis. The latter include cytosolic acidification, calcium levels, endoplasmic reticulum stress signaling pathways and reactive oxygen species (ROS) production^19^. In fact, BI-1 interaction with ROS production is highly relevant for corals under thermal stress, as accumulation of ROS has been suggested to be a major trigger of apoptosis, giving rise to coral bleaching and subsequent death^26^. BI-1 anti-apoptotic action initially enables the cell to adapt to stress^19^, and therefore the over-expression of the BI-1 gene during the first days of stress could promote cell survival and the down-regulation of apoptosis.

The induction of apoptosis in the first hours/days of stress is evident by the significant changes that occur in caspase activity and in the expression levels of the *Sty*Casp gene (Fig. 1C,B). It is further corroborated by the significant up-regulation of the apoptotic protease activating factor 1 (APAF-1) at 6–48 hts, with a fold increase of 2.4–3 (Fig. 1F), coinciding with the up-regulation of the *Sty*Casp gene and with caspase activity. APAF-1 is a cytoplasmic protein that plays a central role in the apoptotic regulatory network. Upon binding cytochrome C this protein forms an oligomeric apoptosom, essential to the activation of caspases^16^,^17^ (Fig. 1A).

The anti-apoptotic/pro-cell survival survivin gene expression profile (Fig. 1I), unlike those of other target genes studied herein, exhibited a robust (15-fold) increase at 48 h and was still significantly high (3.5 fold) at 72 hts. Survivin is a unique member of the highly conserved inhibitor of apoptosis protein (IAPs) family. The survivin pathway interfaces with the cell-death machinery by inhibiting caspase activity, and also with mechanisms of cell-cycle progression and microtubule stability. The up-regulation of survivin is considered critical for the prevention of the cell death cycle^27^ (Fig. 1A). Interestingly, in *A*. *aspera*, a coral species that dies after thermal stress and bleaching, significant down-regulation of survivin was reported at the bleaching threshold^13^ prior to coral death. The temporally shifted gene expression pattern of survivin appears to reflect its downstream position in the apoptotic cascade, contrary to the *Sty*Bcl-2 and Bax expression patterns (Fig. 1), further aiding in the acclimation of the coral to the sustained stress.

In view of the above, our results corroborate the “two- stage” response model in corals resistant to thermal stress. The first stage (6–48 hts) involves the onset of apoptosis in some cells, as indicated by the up regulation of caspase, Bax and APAF-1, as well as by caspase activity. Concomitantly, cascades of anti-oxidant/anti-apoptotic mediators such as HSP70, Bcl-2 and BI-1 are being up-regulated as “first aid” in order to block the progression of apoptosis in other cells. This acute response is already clearly evident at 6 hts, but could begin earlier, even at temperatures below the bleaching threshold, as observed in *A*. *aspera*^13^. Moreover, time shifts between the pro- and the anti-apoptotic responses could exist (i.e., the pro-apoptotic response occurs first, followed by an immediate anti apoptotic response), but a more refined time-tuned sampling (e.g., every 4 hours) might be necessary if one is to address this question. The second stage of the model (starting at 48–72 hts) could include not only an abatement of the apoptosis-related response (as evidenced by the down regulation of the caspase activity and the expression of caspase, Bax, APAF-1, HSP70, Bcl-2 and BI-1), but also the up-regulation of additional anti–apoptotic/pro-cell survival regulators (as indicated by the robust expression of servivin) that could fill an important role in the acclimation of the coral to the sustained stress. Yet, the functional characterization of these genes is essential to support this model.

### A functional analysis of the coral Bcl-2 family member *Sty*Bcl-2

**Figure 2:**
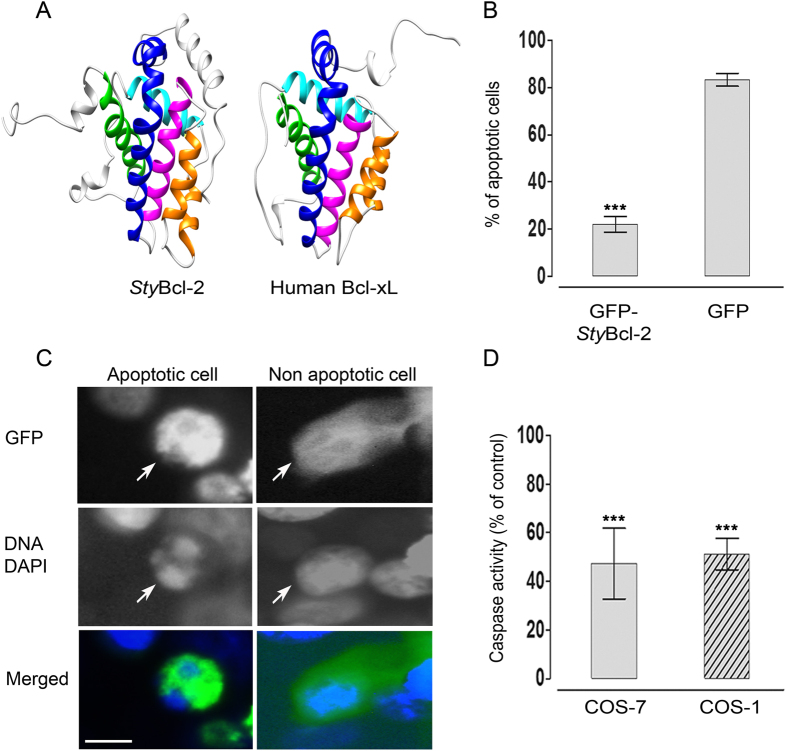
*Sty*Bcl-2 inhibits apoptosis in mammalian cell lines. (**A**) The predicted three dimensional structure of *Sty*Bcl-2 (accession No. EU715319.1), as compared to human anti-apoptotic Bcl-xL (accession No. 1YSG_A). Similar domains are assigned the same color; the BH domains are indicated by green (BH4), cyan (BH3), magenta (BH1) and blue (BH2). (**B**,**C**) COS-7 cells were transfected with GFP (control) or GFP-*Sty*Bcl-2 and treated with staurosporin before analysis. (**B**) Counts of GFP-positive cells with apoptotic morphology expressed as a percentage of all GFP-positive cells. (**C**) Epifluorescence imaging; the left hand panels show a cell undergoing apoptosis, as indicated by its round shape (see GFP, in green in the merged image) and pycnotic chromatin (see DNA stain DAPI, in blue in the merged image); the right hand panel shows a non-apoptotic cell with GFP in the cytoplasm (in green in the merged image), with normal interphase nuclei (see DNA stain DAPI, in blue in the merged image); Scale bar* *= 10 μm. (**D**) Caspase 3 activity was assayed in COS-1 and COS-7 cells transfected with pcDNA3 (control) or pcDNA3-*Sty*Bcl-2 and treated with staurosporin before analysis. Data are standardized as changes relative only to the untreated vector. Results are expressed as the means ± SE of 4 independent essays, each with five replicates per treatment. Values were tested using a One-Way ANOVA and a t-test. Asterisks indicate significant differences between the control and the treatments (***represents P < 0.001).

To facilitate support of the hypothesized anti-apoptotic involvement of coral Bcl-2 proteins in down-regulating the apoptotic response in corals under thermal stress (i.e., first stage of the model), we characterized *Sty*Bcl-2 both structurally and functionally. The homology of *Sty*Bcl-2 to well-studied Bcl-2 family proteins^16^,^17^,^18^ was analyzed by modeling its three dimensional structure (3D). The predicted 3D of *Sty*Bcl-2 showed the highest score with the 3D of the human anti-apoptotic protein Bcl-xL (Fig. 2A). Thus, 88% of the *Sty*Bcl-2 sequence was modeled with 100% confidence to human Bcl-xL by the single highest scoring template. Furthermore, transfected mammalian cell lines (i.e., COS-1 and COS-7) that transiently expressed *Sty*Bcl-2 were studied qualitatively and quantitatively in order to verify the role of the coral’s protein in apoptosis. First, a chimeric construct containing the *Sty*Bcl-2 encoding sequence fused to an enhanced green florescent protein (GFP) reporter gene was transfected into COS-7 cells. Then apoptosis was induced with the mitochondrial stressor staurosporine, and the abundance of cells with apoptotic morphology (i.e., with pycnotic chromatin and rounded, shrunken cell bodies, Fig. 2C) was recorded in the GFP-positive cells. The percentage of apoptotic-positive cells was significantly lower (P < 0.05) among the GFP-*Sty*Bcl-2 expressing cells (22.1%) than in control cells that expressed GFP only (83.25%) (Fig. 2B and Table 2). These results are in agreement with the qualitative functional analysis carried out using the Bcl-2 family members from *Hydra vulgaris*^20^ and *Acropora millepora*^21^. In both studies, the transiently-expressed GFP-conjugated Bcl-2-like proteins demonstrated an inhibitory effect on camptothecin-induced (*H*. *vulgaris*) or tBid-triggered (*A*. *millepora*) apoptosis in mammalian cells, as evident by apoptotic cell counts.

To better assess the anti-apoptotic capabilities of *Sty*Bcl-2, and to avoid possible distortion by the conjugated GFP, a plasmid construct containing merely the *Sty*Bcl-2 encoding sequence was transfected into COS-7 and COS-1 mammalian cells, and caspase activity was monitored in each cell type after induction of apoptosis with the mitochondrial stressor staurosporine. Both *Sty*Bcl-2 expressing cell types showed a significantly (P < 0.05) reduced staurosporin-induced caspase activity (50.15% and 55.67% inhibition, COS-7 and COS-1, respectively) compared to the controls (Fig. 2D and Table 2). The results presented herein establish the anti-apoptotic role of *Sty*Bcl-2 and provide fundamental support to the suggested involvement of Bcl-2 in down-regulating the apoptotic response in corals under thermal stress^4^,^14^ and reduced pH conditions^15^.

Altogether our results support the apoptotic “two stage response” model^4^,^7^,^12^,^14^, emphasizing the substantiality and complexity of the apoptotic pathway in the response of corals to global warming and ocean acidification^4^,^7^,^12^,^13^,^14^,^15^, while also corroborating the similarity between the apoptotic networks of simple metazoans and the networks of higher animals^13^,^18^,^20^,^21^.

## Materials and Methods

### Experimental design, sample collection, sample processing, quantitative PCR (qPCR) and statistical analyses

The samples used for qPCR in this study were taken from experiment no. 3^4^. In short, coral fragments of *S. pistillata* were subjected to thermal stress (34 °C) or control (24 °C) conditions for 168 h. Bleaching was observed after 72 h of stress, and the bleached corals survived up to one month at thermal stress. When placed at ambient temperature, the bleached corals slowly recovered, recovering back symbionts^4^. Six fragments from each aquarium were sampled at time points of 0, 6, 24, 48, 72 and 168 h and snap-frozen with liquid nitrogen. RNA and protein from each fragment were extracted and quantified as described^4^. qPCR primers (Table 3) were designed using primer express 3.0 (Applied Biosystems, Foster City, USA), and qPCR was carried out as described^4^. The copy number of the transcripts for each gene in unknown samples was determined by comparing CT values of each gene with the internal control gene adenosyl-homocysteinase (AdoHcyase) from *S*. *pistillata* host tissue, as decribed^4^. A caspase activity assay and a qPCR for *Sty*Casp and *Sty*Bcl-2 were reported previously^4^. Statistical significance was tested using a Two-Way Anova with Tukey post-hoc testing (Table 1). The analyses were performed using the JMP (ver. 7.0.1) statistical software (SAS Institute Inc., Cary, NC).

### Sequence identification and three dimensional structure (3D) models

To make the current study more comprehensive, we included the expression profiles of the *Sty*Casp and *Sty*Bcl-2 genes as well as the caspase activity reported in our previous study^4^ in Fig. 1 and in the discussion. The cDNA sequences of the Bcl-2 family member Bak, APAF-1 and the key downstream inhibitors of apoptosis, BIR (survivin) and BI-1 (Bax inhibitor-1), were all identified based on data from the Centre Scientifique de Monaco (http://data.centrescientifique.mc/CnidBar-home.html) and UniPort (http://www.uniprot.org/uniprot), based on sequence homology within evolutionarily conserved domaines to *A*. *aspera* genes^13^. The regulators and inhibitors of apoptosis were aligned and compared to previously identified apoptosis regulators^13^ using the NCBI blast server. qRT–PCR primers (Table 3) were designed using the primer express 3.0 software (Applied Biosystems, Foster City, USA). To confirm the accuracy of the primers and correct identification of the genes in *S*. *pistillata*, the qPCR products were sequenced and their homology to higher organism cell death genes as well as to the published sequences of *A*. *aspera*^13^ was confirmed. The HSP70 sequence was downloaded from Genbank (Accession AF152004).

The predicted 3D model of *Sty*Bcl-2 (accession No. EU715319.1) was created based on multiple-threading alignments and iterative assembly simulations using the protein homology/analogy recognition engine (Phyre, http://www.sbg.bio.ic.ac.uk/~phyre2/) and the RCSB protein data bank (PDB, http://www.rcsb.org/pdb/)^28^. The resulting 3D model of *Sty*Bcl-2 was aligned with the 3D of the highest scoring template, which was that of Bcl-xl (accession No. 1YSG_A). Images of molecular graphics were produced using the UCSF Chimera package 1.10. from the Resource for Biocomputing, Visualization, and Informatics at the University of California, San Francisco (supported by NIH P41 RR-01081)^29^.

### Plasmid construction

Commercially available gene synthesis (GenScript) was used to generate the open reading frame that encodes for *Sty*Bcl-2^4^. The latter sequence was then cloned into pcDNA3.1 (Clontech, CA, USA) downstream of the gene that encodes the green fluorescence protein (GFP), giving rise to a translational fusion of N-terminal GFP-tagged *Sty*Bcl-2 proteins.

### Cell culture, transfection and induction of apoptosis in mammalian cells

African green monkey kidney cell lines (COS-1 and COS-7 cells; Invitrogen, Carlsbad, CA) were cultured in MEM – ALPHA (COS-1) or DMEM (COS-7) (Sigma, MO, USA), supplemented with 10% FCS and 1% Pen-strep-Ampho. At 70% cell confluence, the cells were harvested using 0.25% Trypsin EDTA (Sigma, MO, USA), plated in 24-well flat-bottom sterile microplates (Sigma, MO, USA) at a density of 40,000 cells/well and grown overnight at 37 °C under 5% CO_2_. Transient transfections of COS cells with 0.7 μg of the pcDNA- StyBcl-2 vector (treatment) or with 0.7 μg of the pcDNA vector (control) were performed, using the FuGene 6 transfection reagent (Roche Diagnostics, Basel, Switzerland) according to the manufacturer’s instructions. Each experimental group consisted of 5 replicates (5 wells in the 24 well-plate). The transfected cells were maintained for 24 h in an incubator at 37 °C and 5% CO_2_. To induce apoptosis, cells were treated with 2 μm staurosporin (Sigma, MO, USA) for 4 h (COS-7) or 1 μm staurosporin for 8 h (COS-1). Both floating and adherent cells were harvested using the Cell Culture Lysis Reagent (Promega Madison, WI, USA) and pooled. Each replicate was analyzed for caspase activity^4^, and protein content was measured using the BCA Protein Assay Kit (Pierce). This procedure was repeated at least four independent times for each cell type.

### Apoptotic nuclei counts

COS-7 cells were cultured and harvested as described above, then plated on poly-lysine-coated glass coverslips at a suitable dilution in 24-well flat-bottom sterile microplates (Greiner) and grown overnight at 37 °C under 5% CO_2_. Cells were transfected with 0.7μg of either pEGFP-StyBcl-2 (GFP-StyBcl-2) or pEGFP vector (GFP, control), using the FuGene 6 transfection reagent (Roche Diagnostics, Basel, Switzerland) according to the manufacturer’s instructions. Cells were grown overnight as described above and apoptosis was induced through treatment with 2 μm of staurosporin (Sigma, MO, USA) for 4 h. Cells were fixed in 4% PFA, washed in PBS and counterstained with 0.1 mg/ml 49,6-diamidino-2-phenylindole (DAPI, Sigma, MO, USA). The nuclei of GFP-expressing cells were microscopically assayed for apoptotic morphology and the percentage of apoptotic nuclei was counted. Each treatment had a minimum of 4 counts (with a minimum of 100–200 cells/count). Photomicrographs were taken using an Epifluorescence microscope (Olympus BX51) and a Scion camera (Color IEEE-1394). The grayscale single channel images were overlaid on an RGB image, assigned a false color to each channel and then reassembled using the Adobe Photoshop 7.0 software (Adobe Systems, USA).

### Statistical analyses

Statistical significance was tested using a One-Way ANOVA and a t-test (Table 2). The analyses were performed using the JMP (ver. 7.0.1) statistical software (SAS Institute Inc., Cary, NC).

## Additional Information

**How to cite this article**: Kvitt, H. *et al*. The regulation of thermal stress induced apoptosis in corals reveals high similarities in gene expression and function to higher animals. *Sci. Rep.* **6**, 30359; doi: 10.1038/srep30359 (2016).

## Figures and Tables

**Table 1:** Significant gene expression changes for target genes of interest and significant caspase activity changes through the thermal stress period. hts, hours of thermal stress.

Gene	6 hts	24 hts	48 hts	72 hts
*Sty*Casp	1.17	1.78	1.24	
	*p* < 0.0001	*p* < 0.0001	*p* < 0.0001	
*Sty*Bcl-2	1.39	1.26	0.67	
	*p* < 0.0001	*p *< 0.0001	*p *= 0.0192	
Bax	2.03	1.77		
	*p* < 0.0001	*p* < 0.0001		
APAF-1	1.42	1.86	1.65	
	*p *= 0.0035	*p *= 0.0047	*p *= 0.0003	
BI-1	0.54	0.51		
	*p* < 0.0001	*p *= 0.0007		
HSP70	1.3			
	*p* < 0.0001			
Survivin			1.82	0.59
			*p* < 0.0001	*p *= 0.0465
Caspase activity	2.68	4.9	3.87	
	*p *= 0.0083	*p* < 0.0001	*p *= 0.0021	

**Table 2:** Significant changes in the percent of caspase activity and of apoptotic nuclei counts in cell lines expressing *Sty*Bcl-2 as compared to controls.

Cell line	COS 7	COS 1
Caspase activity	50.15%	55.67%
	*p *< 0.0001	*p *< 0.0001
apoptotic nuclei counts	22.1%	
	*p *< 0.0001	

**Table 3:** Primer sequences utilized in quantitative PCR.

Gene	Primer direction	Primer sequence 5′-3′
Bax like	F	ATTAAGGACCGCTTGGCAAA
Bax like	R	CCAAATCATGCCTCTGTCTCAA
APAF-1 like	F	AGCAAGTTGGTGTCATCCTCCGAT
APAF-1 like	R	ATAGCAACAAAGCTCTCCGGTGGA
BI-1 like	F	TGGGCAAAGGAGCGATTTTA
BI-1 like	R	AGGTTGGCCAGTGACAGGAA
Hsp70	F	AGGCAACTCTCAACCCAAACA
Hsp70	R	AAAAGTGCGCGTGCAGTACA
Survivin like	F	ACGGAAGAAACGGGATCTTGT
Survivin like	R	CAAAGCAACGTGCAACATCAG
*Sty*Bcl-2^4^	F	CGTCGTGGCCCACCATT
*Sty*Bcl-2^4^	R	CCACGATCTTCTGAACCATTTCT
*Sty*Casp^4^	F	GGACGGCATGGACGTAACAG
*Sty*Casp^4^	R	CAGCTGGGACAGAGACTCGAT
